# The association between HPV vaccination and new-onset cardiovascular and cerebrovascular diseases: based on a retrospective study

**DOI:** 10.1186/s41043-025-00918-y

**Published:** 2025-05-19

**Authors:** Chiao-Yu Yang, Yu-Hsiang Shih, Chia-Chi Lung

**Affiliations:** 1 Department of Occupational Health Nursing Center, Chung Shan Medical University Hospital, Taichung City, Taiwan; 2 Department of Public Health, Institute of Public Health, Chung Shan Medical University, No. 110, Sec. 1 Jianguo N. Rd., Taichung City, 40201 Taiwan; 3 Department of Obstetrics and Gynecology, Taichung Veterans General Hospital, Taichung City, Taiwan; 4 Department of Health Policy and Management, Chung Shan Medical University, Taichung City, Taiwan; 5 Department of Family and Community Medicine, Chung Shan Medical University Hospital, Taichung City, Taiwan

**Keywords:** HPV vaccination, Cardiovascular diseases, Cerebrovascular diseases, Heart dysfunction, Public health, Immunization and heart health

## Abstract

**Introduction:**

Human papillomavirus (HPV), a widespread sexually transmitted infection, is well-known for its association with cancers like cervical, anal, and oropharyngeal. While extensively studied for its oncogenic properties, HPV’s influence on these non-cancerous conditions remains under investigation. This study examines the impact of HPV vaccination on the incidence of cardiovascular and cerebrovascular diseases, exploring its protective potential.

**Methods:**

Utilizing the global TriNetX database, this retrospective cohort study analyzed data from adults aged 20 to 40 who were either vaccinated or not vaccinated against HPV between January 1, 2019, and December 31, 2021. The study employed propensity score matching to ensure balanced comparison groups and utilized ICD-10 codes to identify new-onset cardiovascular and cerebrovascular diseases.

**Results:**

The study included 59,423 vaccinated and an equal number of unvaccinated individuals, balanced for various demographic and health characteristics. The vaccinated group demonstrated a lower incidence of cardiovascular diseases (hazard ratio [HR] = 0.9, 95% CI 0.847–0.957), cerebrovascular diseases (HR = 0.605, 95% CI 0.484–0.754), and heart dysfunction (HR = 0.833, 95% CI 0.708–0.98) compared to the unvaccinated group. Subgroup analyses further supported the protective effect of HPV vaccination across different demographics.

**Conclusion:**

HPV vaccination is linked to a lower risk of new-onset cardiovascular and cerebrovascular diseases in adults, suggesting broader health benefits beyond cancer prevention. These results advocate for incorporating cardiovascular health into public health strategies promoting HPV vaccination. Further research is necessary to understand the mechanisms and validate these protective effects across diverse populations and extended follow-up periods.

**Supplementary Information:**

The online version contains supplementary material available at 10.1186/s41043-025-00918-y.

## Introduction

Human papillomavirus (HPV), a DNA virus, is one of the most common sexually transmitted infections globally, affecting millions each year. There are over 200 different types of HPV, with some causing benign conditions like warts and others being linked to various cancers, including cervical, anal, and oropharyngeal cancers. [1, 2] Additionally, HPV infection has drawn attention due to its potential association with non-cancerous conditions, particularly cardiovascular and cerebrovascular diseases. Recent studies have expanded the understanding of HPV’s impact beyond oncogenesis, suggesting that HPV may also play a role in the development of cardiovascular disease (CVD) [3, 4]. Given the extensive research on HPV’s oncogenic properties, a natural progression of inquiry leads us to explore its potential impacts on cardiovascular health, a relatively underexplored domain. A review study indicated a link between vaginal HPV infection and cardiovascular diseases (odds ratio = 1.66, 95% CI 1.28–2.16). [3] In the United States, a cross-sectional study found that women with genital HPV infection, especially high-risk HPV (HR-HPV), had nearly three times the history of myocardial infarction or stroke compared to those without HPV. [5] Another cross-sectional study suggested a potential association between HPV infection and coronary artery disease (CAD) in menopausal women [6]. A 17-year cohort study in Korean women revealed that those infected with high-risk HPV had a significantly increased risk of death from atherosclerotic cardiovascular disease (ASCVD) and ischemic heart disease (IHD) [7]. Regarding cerebrovascular health, HPV-positive status is significantly correlated with an elevated risk of stroke or transient ischemic attack in patients undergoing radiation therapy for head and neck cancer [8]. These findings suggest that HPV infection may contribute to a broader spectrum of health issues, particularly in populations already at risk for cardiovascular events.

Viral infections contribute to cardiovascular disease through multiple mechanisms. It is well-documented that viral pathogens can play a critical role in the initiation and progression of atherosclerosis [9, 10]. They can initiate and exacerbate atherosclerosis by promoting chronic inflammation, endothelial injury, and lipid accumulation. Viruses like CMV and HIV disrupt normal vasomotor function, induce procoagulant states, and increase levels of matrix metalloproteinases (MMPs), leading to plaque instability and rupture [11, 12]. Additionally, infections can trigger systemic inflammation, further aggravating atherogenesis. The process involves complex immune responses, including T-cell activation and the production of pro-inflammatory cytokines, which ultimately contribute to plaque progression and cardiovascular events. [13]

HPV vaccines can prevent certain infections and are a key public health intervention for reducing HPV-associated diseases. In the United States, available vaccines include Cervarix (approved in 2009), Gardasil (2006), and Gardasil 9 (2014, for both sexes). Vaccination is recommended for individuals under 26 years, with those aged 27–45 advised to consider it based on personal risk factors. This backdrop of emerging research sets the stage for our study, aiming to directly investigate the potential cardiovascular benefits of HPV vaccination. A recent cross-sectional study indicated that there is an association between HPV infection and cardiovascular diseases, though this association is not significant among vaccinated women [14]. Nevertheless, there is limited literature exploring the association between HPV vaccines and the incidence of new-onset of cardiovascular and cerebrovascular diseases.

This study aims to explore whether HPV vaccination is linked to an reduce risk of developing new-onset cardiovascular and cerebrovascular diseases. By leveraging the extensive data available in the TriNetX database, we can conduct a comprehensive analysis to identify any significant patterns or correlations. Such research could provide crucial insights into the broader benefits of HPV vaccination, potentially influencing public health strategies and vaccine recommendations.

## Methods

### Data source

This study utilized the TriNetX database, a global health research network containing de-identified electronic medical records from approximately 250 million individuals across 120 healthcare organizations. The database includes comprehensive data on demographics, diagnoses (ICD-10-CM), procedures (ICD-10-PCS or CPT codes), medications (Veterans Affairs National Formulary), laboratory measurements (LOINC-coded), and healthcare utilization.

Participants were adults from the TriNetX US Collaborative Network, a real-time, multicenter national health network comprising data from 64 healthcare organizations across all 50 U.S. states. This network provides a diverse and representative sample of the U.S. population, ensuring the generalizability of the study’s findings. Data analysis was conducted in August 2024, covering the period from January 1, 2019, to December 31, 2021, allowing for the assessment of healthcare trends during this timeframe.

### Study design

**Fig. 1:**
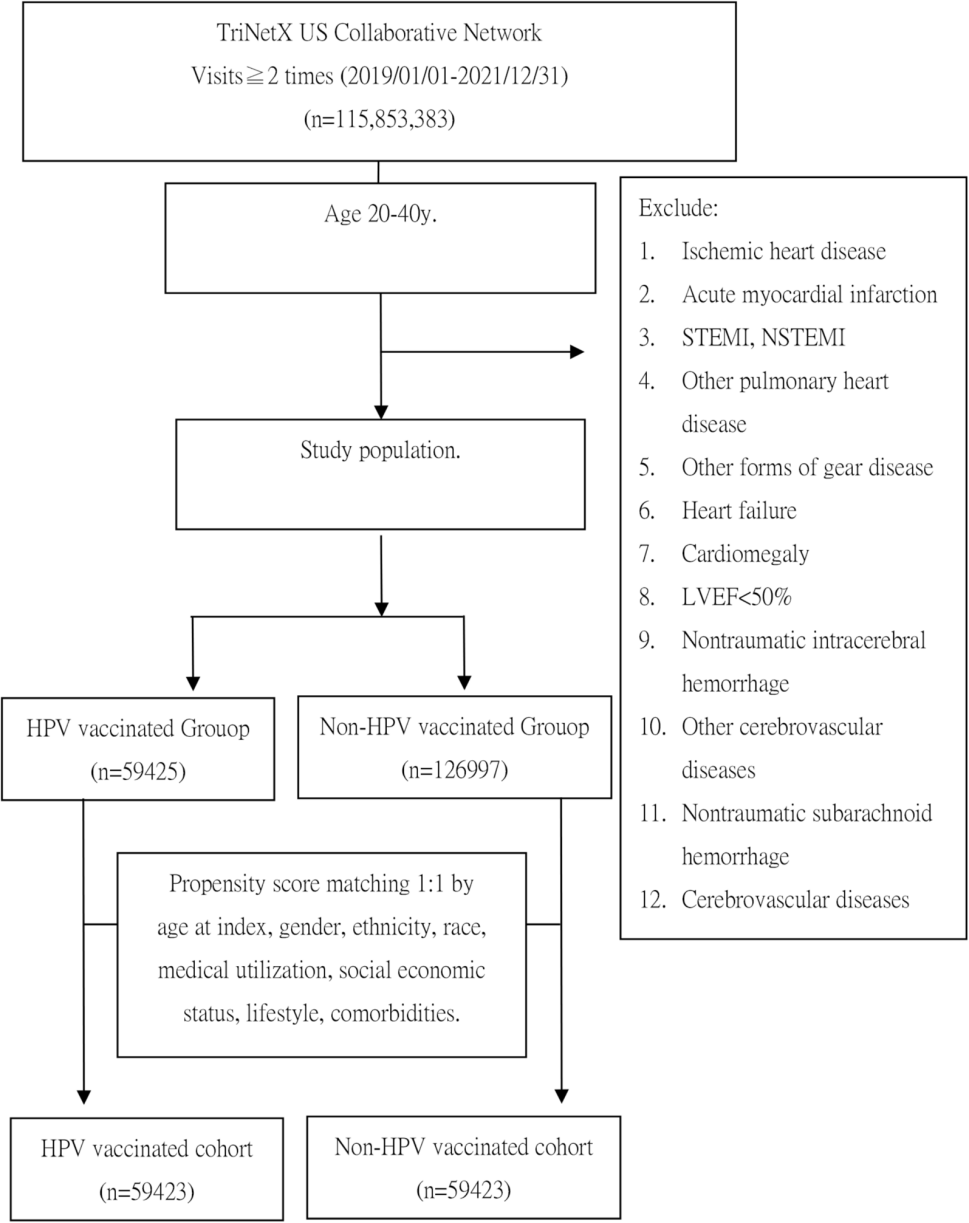
Study flowchart

This retrospective cohort study utilized data from the TriNetX database, a comprehensive global health research network that provides access to de-identified electronic medical records from various healthcare institutions. The primary aim of the study was to investigate the association between HPV vaccination and the incidence of new-onset cardiovascular and cerebrovascular diseases in adults. Figure 1 illustrates the cohort selection process, which included individuals aged 20–40 who either received or did not receive the HPV vaccine between January 1, 2019, and December 31, 2021. The index date was defined as the date of HPV vaccination for individuals who were vaccinated, and as the date of a general examination for those who were unvaccinated.

Baseline characteristics and comorbidities were assessed using medical records from the year preceding the index date. These included demographics (age, gender, BMI, ethnicity, and race), healthcare utilization (outpatient/inpatient services), socioeconomic factors (e.g., housing challenges, healthcare access, and employment), and lifestyle factors (e.g., nicotine dependence, alcohol disorders). Comorbidities such as hypertension, diabetes, liver disease, CKD, obesity, and hyperlipidemia were also considered. We applied Propensity Score Matching (PSM), a statistical technique to ensure comparable groups by balancing variables that could introduce selection bias, thus enhancing the study’s reliability. Matching was conducted using a 1:1 ratio, and balance was confirmed with Standardized Mean Differences (SMDs) below 0.1, ensuring comparability between groups.

### Outcomes

The three major outcomes of this study were the incidences of new-onset cardiovascular diseases, cerebrovascular diseases, and heart dysfunction. These outcomes were identified using ICD-10 codes from the database. Specifically, cardiovascular diseases were defined primarily using codes I20–I25 (ischemic heart diseases) and I50 (heart failure); cerebrovascular diseases using codes I60–I69 (including hemorrhagic and ischemic stroke); and heart dysfunction using codes reflecting systolic, diastolic, and combined heart failure. To mitigate the effects of competing risks and survivorship bias, a combination of ICD-10 codes was also used to define composite outcomes. All cases were followed for a period of 3 years. A complete list of codes used to define covariates and outcomes is provided in Supplementary Table S1.

### Subgroup analyses

Subgroup analyses were conducted using the TriNetX network to explore how sex, age, and race influence the risk of new-onset cardiovascular diseases, cerebrovascular diseases, and heart dysfunction. Participants were stratified by sex (female and male), age groups (20–30 years and 30–40 years), and race (White, Black or African American, Asian, and other races). This approach provided a detailed examination of potential variations in risk across these demographic factors.

### Sensitivity analyses

The sensitivity analyses utilized varying follow-up periods, from index date to year 5 post-index date, to test the reliability of the study’s outcomes.

### Statistical analyses

Data management and HRs calculations with 95% confidence intervals (CIs) were conducted on the TriNetX platform. PSM achieved a 1:1 match based on variables such as age, sex, race, socioeconomic status, and comorbidities, ensuring comparability between groups with a standardized mean difference (SMD) below 0.1. HRs were calculated using the Cox proportional hazards model, and statistical significance was determined via the log-rank test (*p* < 0.05). Kaplan–Meier curves were used to assess event rates over time and compare survival outcomes between groups.

To address potential biases and confounding, PSM was utilized to align participants with similar exposure probabilities, thereby reducing bias. The effectiveness of the matching process was assessed by examining any residual imbalances, ensuring that this method effectively controlled for confounding variables.

## Results

### Baseline characteristics of the study individuals

In this study, 59,425 individuals received the HPV vaccine, while 126,997 individuals did not (Table 1). After PSM, both groups comprised 59,423 individuals, ensuring the balance of baseline characteristics. Prior to matching, the HPV vaccine group had a higher proportion of females and a younger average age of 25.8 years, compared to 30.6 years in the non-HPV vaccine group. After matching, the gender distribution and age were consistent between the two groups, demonstrating good balance. Additionally, medical utilization rates and other socioeconomic factors were highly comparable between the groups, with standardized mean differences (SMDs) all below 0.1, indicating that any differences in baseline characteristics were effectively controlled. This ensured the reliability of the analysis results.

**Table 1:** Baseline characteristics

	Before PSM					After PSM				
	HPV vaccine		Non-HPV vaccine		SMD	HPV vaccine		Non-HPV vaccine		SMD
	N	%	N	%		N	%	N	%	
No. of individuals	59,425		126,997			59,423		59,423		
*Sex*										
Female	40,964	68.93%	717,290	56.48%	0.2597	40,963	68.94%	40,866	68.77%	0.0035
Male	16,227	27.31%	471,589	37.13%	0.2114	16,226	27.31%	16,329	27.48%	0.0039
Age at index, years (mean ± SD)	25.8 ± 5.25		30.6 ± 6.02		0.8362	25.8 ± 5.25		25.8 ± 5.23		0.0087
BMI (mean ± SD)	27.8 ± 7.53		27.8 ± 7.53		0.1848	27.8 ± 7.53		28.4 ± 7.7		0.0719
*Ethnicity*										
Hispanic or Latino	11,782	19.83%	120,718	9.51%	0.2949	11,780	19.82%	11,707	19.70%	0.0031
Not Hispanic or Latino	39,148	65.88%	841,748	66.28%	0.0085	39,148	65.88%	39,388	66.28%	0.0085
*Race*										
Asian	4680	7.88%	70,515	5.55%	0.0929	4678	7.87%	4784	8.05%	0.0066
White	34,131	57.44%	746,233	58.76%	0.0268	34,131	57.44%	34,067	57.33%	0.0022
Black or African American	9047	15.22%	176,048	13.86%	0.0386	9047	15.23%	9108	15.33%	0.0029
Unknown Race	7397	12.45%	203,192	16.00%	0.1018	7397	12.45%	7399	12.45%	0.0001
Other Race	3698	6.22%	65,423	5.15%	0.0463	3698	6.22%	3611	6.08%	0.0061
*Medical utilization*										
Office or Other Outpatient Services	36,248	61.00%	459,031	36.14%	0.5134	36,246	61.00%	35,727	60.12%	0.0179
Hospital Inpatient and Observation Care Services	1698	2.86%	21,552	1.70%	0.0778	1698	2.86%	1505	2.53%	0.0201
*Social economic status*										
Problems related to housing and economic circumstances	41	0.07%	371	0.03%	0.0180	41	0.07%	26	0.04%	0.0106
Factors influencing health status and contact with health services	39,216	65.99%	547,070	43.08%	0.4729	39,214	65.99%	39,036	65.69%	0.0063
Problems related to employment and unemployment	86	0.15%	1564	0.12%	0.0059	86	0.15%	65	0.11%	0.0099
Problems related to education and literacy	31	0.05%	220	0.02%	0.0187	31	0.05%	21	0.04%	0.0080
*Lifestyle*										
Nicotine dependence	1766	2.97%	34,134	2.69%	0.0171	1766	2.97%	1692	2.85%	0.0074
Alcohol related disorders	411	0.69%	9178	0.72%	0.0037	411	0.69%	353	0.59%	0.0122
*Comorbidities*										
Essential (primary) hypertension	1551	2.61%	49,268	3.88%	0.0717	1551	2.61%	1379	2.32%	0.0187
Mental, Behavioral and Neurodevelopmental disorders	14,164	23.84%	220,939	17.40%	0.1597	14,164	23.84%	13,961	23.49%	0.0080
Neoplasms	2787	4.69%	47,196	3.72%	0.0485	2787	4.69%	2617	4.40%	0.0137
Pregnancy, childbirth, and the puerperium	5362	9.02%	58,599	4.61%	0.1756	5362	9.02%	4895	8.24%	0.0280
Type 2 diabetes mellitus	763	1.28%	17,257	1.36%	0.0066	763	1.28%	649	1.09%	0.0177
Endocrine, nutritional and metabolic diseases	11,166	18.79%	174,981	13.78%	0.1361	11,165	18.79%	10,807	18.19%	0.0155
Cardiac arrhythmia	10	0.02%	76	0.01%	0.0102	10	0.02%	10	0.02%	0.0000
Diseases of liver	429	0.72%	10,819	0.85%	0.0147	429	0.72%	347	0.58%	0.0171
Chronic kidney disease (CKD)	110	0.19%	2987	0.24%	0.0109	110	0.19%	65	0.11%	0.0198
Rheumatoid arthritis, unspecified	57	0.10%	1561	0.12%	0.0082	57	0.10%	49	0.08%	0.0045
Sleep disorders	1841	3.10%	35,534	2.80%	0.0177	1841	3.10%	1735	2.92%	0.0104
Overweight and obesity	5836	9.82%	68,637	5.41%	0.1671	5835	9.82%	5606	9.43%	0.0131
Hyperlipidemia, unspecified	569	0.96%	17,806	1.40%	0.0412	569	0.96%	496	0.84%	0.0130
Systemic connective tissue disorders	239	0.40%	4577	0.36%	0.0068	239	0.40%	178	0.30%	0.0174

SD, standard deviation; SMD, standardized mean difference; PSM: matching factors included sex, age at index, BMI, ethnicity, race, medical utilization, social economic status, and comorbidities

### Incidence of outcomes in the HPV vaccinated and Non-HPV vaccinated groups

As shown in Table 2, the incidence of cardiovascular diseases was lower in the HPV-vaccinated group than in the non-vaccinated group (HR 0.9, 95% CI 0.847–0.957), with survival probabilities of 95.678% versus 95.26% at the end of follow-up. Similarly, the vaccinated group had a significantly reduced risk of cerebrovascular diseases (HR 0.605, 95% CI 0.484–0.754), with survival probabilities of 99.706% versus 99.528%. For heart dysfunction, the vaccinated group showed a reduced risk (HR 0.833, 95% CI 0.708–0.98) and survival probabilities of 99.386% versus 99.274%.

**Table 2:** HR and 95% CIs for the risk of outcomes (n = 59,423), follow for 3 years

Outcome	Cohort		
	Patients with outcome	Survival probability at end of time window	HR (95% CI)
*1. Cardiovascular diseases*			
HPV vaccine	1935	95.678%	0.9 (0.847, 0.957)
Non-HPV vaccine	2168	95.26%	Reference
*2. Cerebrovascular diseases*			
HPV vaccine	125	99.706%	0.605 (0.484, 0.754)
Non-HPV vaccine	210	99.528%	Reference
*3. Heart dysfunction*			
HPV vaccine	266	99.386%	0.833 (0.708, 0.98)
Non-HPV vaccine	324	99.274%	Reference
*4. Composite outcome*			
HPV vaccine	2024	95.471%	0.878 (0.827, 0.932)
Non-HPV vaccine	2324	94.915%	Reference

Hazard ratio (HR) and 95% CI are provided

**Fig. 2:**
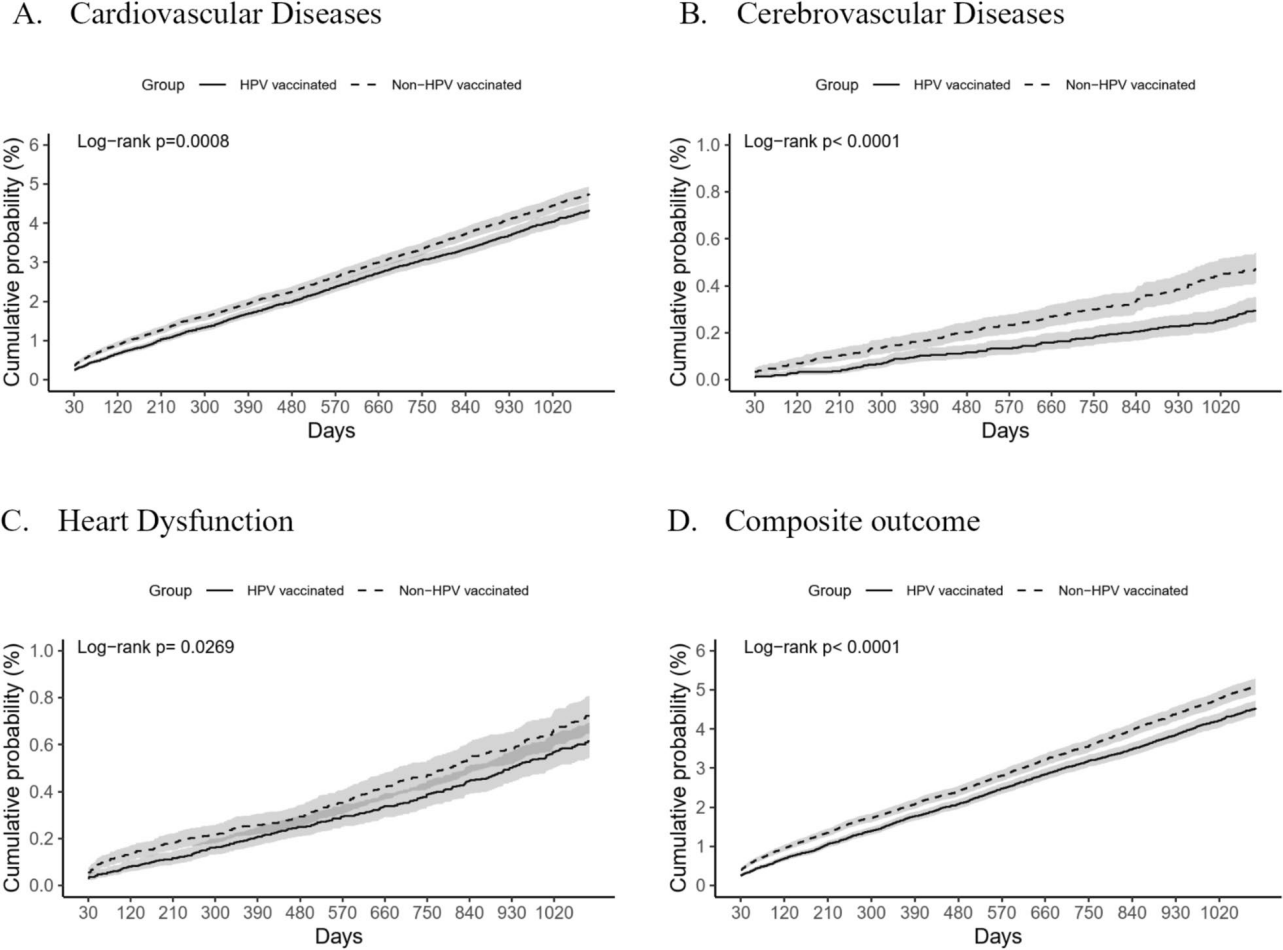
Kaplan–Meier curves illustrating cumulative probability (%) of health outcomes from day 30 to year 3 post-index date, comparing HPOV vaccinated and non-vaccinated groups: **A** cardiovascular diseases, **B** cerebrovascular diseases, **C** heart dysfunction, **D** composite outcome

The composite outcome, including cardiovascular events, cerebrovascular events, or heart dysfunction, also favored the vaccinated group (HR 0.878, 95% CI 0.827–0.932), with survival probabilities of 95.471% versus 94.915%. Kaplan–Meier curves (Fig. 2) illustrate significant differences between the groups (log-rank test, *p* < 0.001).

Table 3 provides HR and 95% confidence intervals for these outcomes over a 3-year follow-up period, excluding individuals with prior HPV infection. The HPV-vaccinated group consistently showed lower risks, though the reduced risk for heart dysfunction was not statistically significant.

**Table 3:** Hazard Ratios (HR) and 95% Confidence Intervals (CI) for the Risk of Outcomes Over 3 Years (n = 51,922), Excluding Individuals with Prior HPV Infection (ICD Codes: B97.7, 87,624, 87,625)

Outcome	Cohort		
	Patients with outcome	Survival probability at end of time window	HR (95% CI)
*1. Cardiovascular diseases*			
HPV vaccine	1794	95.432%	0.907 (0.851, 0.967)
Non-HPV vaccine	1996	95.012%	Reference
*2. Cerebrovascular diseases*			
HPV vaccine	97	99.735%	0.643 (0.498, 0.829)
Non-HPV vaccine	153	99.618%	Reference
*3. Heart dysfunction*			
HPV vaccine	255	99.331%	0.88 (0.744, 1.041)
Non-HPV vaccine	294	99.244%	Reference
*4. Composite outcome*			
HPV vaccine	1860	95.252%	0.889 (0.835, 0.946)
Non-HPV vaccine	2110	94.736%	Reference

### Subgroup analysis

In subgroup analysis (Table 4), HPV vaccination reduced the incidence of cardiovascular diseases, cerebrovascular diseases, heart dysfunction, and composite outcomes across cardiovascular diseases, 0.512 (95% CI 0.309–0.848) for cerebrovascular diseases, 0.847 (95% CI 0.653–1.1) for heart dysfunction, and 0.914 (95% CI 0.811–1.03) for the composite outcome. In females, HRs were 0.807 (95% CI 0.749–0.868) for cardiovascular diseases, 0.625 (95% CI 0.481–0.812) for cerebrovascular diseases, 0.679 (95% CI 0.555–0.831) for heart dysfunction, and 0.791 (95% CI 0.736–0.85) for the composite outcome.

**Table 4:** Subgroup analysis

	Cardiovascular Diseases			Cerebrovascular Diseases			Heart Dysfunction			Composite outcome		
	Patients with outcome		HR (95% CI)	Patients with outcome		HR (95% CI)	Patients with outcome		HR (95% CI)	Patients with outcome		HR (95% CI)
	HPV vaccine	Non-HPV vaccine		HPV vaccine	Non-HPV vaccine		HPV vaccine	Non-HPV vaccine		HPV vaccine	Non-HPV vaccine	
*Sex*												
Female (n = 39,578)	1260	1595	0.807 (0.749, 0.868)	90	148	0.625 (0.481, 0.812)	157	238	0.679 (0.555, 0.831)	1323	1706	0.791 (0.736, 0.85)
Male (n = 15,067)	503	521	0.948 (0.839, 1.072)	23	44	0.512 (0.309, 0.848)	105	122	0.847 (0.653, 1.1)	517	555	0.914 (0.811, 1.03)
*Age*												
20–30 (n = 49,563)	1639	1759	0.934 (0.873, 0.999)	81	134	0.609 (0.462, 0.802)	211	247	0.859 (0.715, 1.032)	1699	1859	0.916 (0.857, 0.978)
30–40 (n = 13,595)	500	685	0.767 (0.684, 0.861)	50	67	0.802 (0.556, 1.158)	82	101	0.864 (0.646, 1.157)	534	732	0.767 (0.686, 0.858)
*Race*												
Black or African American (n = 8496)	291	335	0.855 (0.731, 1.001)	24	49	0.485 (0.298, 0.79)	61	81	0.745 (0.535, 1.039)	308	368	0.823 (0.708, 0.958)
White (n = 35,692)	1318	1511	0.886 (0.823, 0.954)	66	113	0.599 (0.442, 0.811)	169	209	0.826 (0.674, 1.011)	1365	1599	0.867 (0.807, 0.932)
Asian (n = 4498)	82	107	0.834 (0.625, 1.112)	12	16	0.822 (0.389, 1.739)	10*	14	0.549 (0.221, 1.36)	88	120	0.798 (0.606, 1.051)
Other race (n = 3504)	111	121	0.895 (0.691, 1.157)	10*	10*	0.492 (0.148, 1.633)	16	19	0.826 (0.425, 1.606)	114	125	0.89 (0.69, 1.147)

Hazard ratio (HR) and 95% CI are provided, demonstrating outcomes among Non-HPV vaccinated individuals versus HPV vaccinated counterparts

*To protect patient privacy, numbers are rounded up to 10. This may impact results, particularly for small cohorts and infrequent outcomes

By age, individuals aged 20–30 years showed moderate reductions, with HRs of 0.934 (95% CI 0.873–0.999) for cardiovascular diseases, 0.609 (95% CI 0.462–0.802) for cerebrovascular diseases, and 0.859 (95% CI 0.715–1.032) for heart dysfunction. The protective effect was stronger in the 30–40 years group, with HRs of 0.767 (95% CI 0.684–0.861) for cardiovascular diseases and 0.767 (95% CI 0.686–0.858) for the composite outcome.

By race, Black individuals showed reduced risks, particularly for cerebrovascular diseases (HR: 0.485, 95% CI 0.298–0.79) and the composite outcome (HR: 0.823, 95% CI 0.708–0.958). White individuals had HRs of 0.886 (95% CI 0.823–0.954) for cardiovascular diseases and 0.599 (95% CI 0.442–0.811) for cerebrovascular diseases. The protective effect for Asian and other races was observed but not consistently significant.

In subgroup analyses stratified by sex (Table 5 and Table 6), consistent associations were observed between HPV vaccination and a reduced risk of cardiovascular and cerebrovascular outcomes, while the associations did not reach statistical significance across all outcomes.

**Table 5:** Subgroup analysis for male

	Cardiovascular diseases			Cerebrovascular diseases			Heart dysfunction			Composite outcome		
	Patients with outcome		HR (95% CI)	Patients with outcome		HR (95% CI)	Patients with outcome		HR (95% CI)	Patients with outcome		HR (95% CI)
	Non-HPV vaccine	HPV vaccine		Non-HPV vaccine	HPV vaccine		Non-HPV vaccine	HPV vaccine		Non-HPV vaccine	HPV vaccine	
*Age*												
20–30 (n = 14,160)	486	503	0.946 (0.835, 1.072)	18	45	0.392 (0.227, 0.677)	93	79	1.157 (0.857, 1.562)	498	539	0.904 (0.8, 1.021)
30–40 (n = 2889)	113	169	0.679 (0.535, 0.861)	10	21	0.393 (0.174, 0.888)	23	36	0.654 (0.387, 1.103)	117	182	0.651 (0.516, 0.822)
*Race*												
Black or African American (n = 2244)	72	81	0.835 (0.608, 1.146)	10	12	0.316 (0.102, 0.978)	19	20	0.896 (0.478, 1.68)	75	89	0.79 (0.581, 1.074)
White (n = 9635)	373	347	1.068 (0.923, 1.237)	17	34	0.497 (0.278, 0.889)	65	58	1.112 (0.781, 1.585)	384	370	1.031 (0.894, 1.189)
Asian (n = 1158)	26	28	0.934 (0.547, 1.592)	10*	10*	0.198 (0.023, 1.695)	10*	10*	0.677 (0.191, 2.401)	26	33	0.789 (0.472, 1.319)
Other race (n = 990)	28	36	0.731 (0.446, 1.197)	10*	10*	0.311 (0.032, 2.987)	10*	10*	0.793 (0.242, 2.6)	28	38	0.692 (0.425, 1.128)

Hazard ratio (HR) and 95% CI are provided, demonstrating outcomes among Non-HPV vaccinated individuals versus HPV vaccinated counterparts

*To protect patient privacy, numbers are rounded up to 10. This may impact results, particularly for small cohorts and infrequent outcomes

**Table 6:** Subgroup analysis for female

	Cardiovascular diseases			Cerebrovascular diseases			Heart dysfunction			Composite outcome		
	Patients with outcome		HR (95% CI)	Patients with outcome		HR (95% CI)	Patients with outcome		HR (95% CI)	Patients with outcome		HR (95% CI)
	HPV vaccine	Non-HPV vaccine		HPV vaccine	Non-HPV vaccine		HPV vaccine	Non-HPV vaccine		HPV vaccine	Non-HPV vaccine	
*Age*												
20–30 (n = 33,415)	1099	1229	0.904 (0.834, 0.981)	59	94	0.639 (0.461, 0.885)	115	151	0.774 (0.608, 0.987)	1143	1301	0.888 (0.82, 0.962)
30–40 (n = 10,393)	372	517	0.764 (0.668, 0.872)	39	72	0.579 (0.392, 0.855)	59	83	0.771 (0.552, 1.076)	399	569	0.743 (0.654, 0.845)
*Race*												
Black or African American (n = 6761)	268	300	0.892 (0.757, 1.052)	22	32	0.689 (0.4, 1.186)	49	66	0.743 (0.513, 1.075)	283	322	0.877 (0.748, 1.029)
White (n = 23,141)	825	953	0.893 (0.814, 0.981)	41	83	0.511 (0.352, 0.743)	86	109	0.819 (0.617, 1.087)	857	1014	0.872 (0.796, 0.955)
Asian (n = 3428)	60	77	0.862 (0.615, 1.208)	11	10*	3.128 (0.996, 9.825)	10*	10*	0.573 (0.172, 1.903)	66	80	0.914 (0.66, 1.267)
Other race (n = 2507)	83	83	0.978 (0.722, 1.326)	10*	10*	0.494 (0.123, 1.974)	11	11	0.98 (0.425, 2.26)	86	88	0.956 (0.71, 1.287)

Hazard ratio (HR) and 95% CI are provided, demonstrating outcomes among Non-HPV vaccinated individuals versus HPV vaccinated counterparts

*To protect patient privacy, numbers are rounded up to 10. This may impact results, particularly for small cohorts and infrequent outcomes

### Sensitivity analysis

Table S2 presents a sensitivity analysis conducted to evaluate the reliability of the study’s findings over different follow-up periods, ranging from Day 30 to Year 5 post-index. The analysis consistently demonstrated a protective effect of HPV vaccination against cardiovascular and cerebrovascular outcomes. The hazard ratios (HRs) and confidence intervals (CIs) remained stable across the various timeframes, indicating the robustness of the observed associations. These results affirm the strength of the study’s conclusions regarding the reduced risk of cardiovascular diseases, cerebrovascular diseases, and heart dysfunction associated with HPV vaccination.

## Discussion

Our study of 59,423 vaccinated and 59,423 non-vaccinated individuals aged 20 to 40 revealed a significant association between HPV vaccination and reduced risks of cardiovascular diseases (HR 0.9, 95% CI 0.847–0.957), cerebrovascular diseases (HR 0.605, 95% CI 0.484–0.754), and heart dysfunction (HR 0.833, 95% CI 0.708–0.98). The protective effect was more pronounced in women, with HRs of 0.807 (95% CI 0.749–0.868) for cardiovascular diseases, 0.625 (95% CI 0.481–0.812) for cerebrovascular diseases, and 0.679 (95% CI 0.555–0.831) for heart dysfunction. Those aged 30–40 experienced a greater reduction in cardiovascular disease risk (HR 0.767, 95% CI 0.684–0.861) compared to individuals aged 20–30 (HR 0.934, 95% CI 0.873–0.999). While subgroup analyses generally demonstrated a consistent protective trend associated with HPV vaccination, not all associations reached statistical significance. For instance, in males, the HR for cardiovascular diseases was 0.948 (95% CI 0.839–1.072), and for heart dysfunction, 0.847 (95% CI 0.653–1.1). Similarly, among individuals aged 20–30 years, the HR for heart dysfunction was 0.859 (95% CI, 0.715–1.032). These nonsignificant findings may reflect smaller sample sizes, lower event rates, or residual confounding within these strata. Therefore, the observed protective effects in these subgroups should be interpreted with caution. Larger cohort studies with greater statistical power are warranted to clarify whether the apparent trends reflect true risk reductions.

Numerous studies have indicated an association between HPV infection and cardiovascular diseases. A prospective cohort study found a significant association between high-risk HPV infection and an elevated risk of cardiovascular disease (HR 1.25, 95% CI 1.03–1.52) in women monitored between 2011 and 2016. The risk was even higher in individuals with obesity (HR 1.73, 95% CI 1.19–2.51) or metabolic syndrome (HR 1.99, 95% CI: 1.28–3.08) [15]. Another study detected HPV gene sequences in 55% of atheromatous coronary arteries from 20 deceased donors, with a significant correlation between HPV E7 protein expression and its presence in smooth muscle cells, foam cells, and macrophages (correlation coefficient = 0.503, *p* = 0.024) [16]. In a study of 52 postmenopausal women, 16 out of 27 with coronary artery disease (CAD) were HPV-positive. After adjusting for various factors, HPV infection, particularly with high-risk types, was strongly associated with an increased risk of CAD (OR = 3.74; 95% CI 1.16 to 11.96; high-risk OR = 4.90; 95% CI 1.26 to 19.08). No link was found between low-risk HPV types and CAD. This study suggests that HPV, along with menopause-related factors like estrogen deficiency and adipose tissue changes, may promote atherosclerosis [6]. Our study, however, focused on a younger group aged 20–40.

There is an increasing body of literature investigating the association between HPV infection and cerebrovascular events. A recent study from South Korea explored this relationship, specifically examining ischemic stroke mortality. The findings revealed that the age-adjusted HR for ischemic stroke mortality in women who tested positive for high-risk HPV was 6.19 (95% CI 0.93–41.11). However, this result was not statistically significant [7]. A retrospective cohort study demonstrated that HPV was detected in 59% of the 326 patients who received radiation therapy for head and neck cancer. During a median follow-up period of 3.4 years, the incidence of cerebrovascular events was significantly higher among HPV-positive patients compared to their HPV-negative counterparts (2.6% vs. 0.9%, *P* = 0.002). Multivariable analysis revealed that HPV-positive status was associated with more than a fourfold increased risk of CVEs (HR 4.4, 95% CI 1.5–13.2, *P* = 0.008) [8]. These findings suggest a potential link between HPV infection and cerebrovascular disease, particularly in high-risk groups like cancer patients. Although the association with ischemic stroke mortality is inconclusive, the increased risk of cerebrovascular events in HPV-positive individuals highlights the need for further research. Understanding this relationship could lead to better prevention and management strategies for affected populations.

New risk factors for cardiovascular disease have been widely studied. The impact of chronic infections may play a significant role in the development, progression, or destabilization of atherosclerotic cardiovascular disease. Research has shown that HPV may contribute to atherosclerosis by infecting vascular or non-vascular cells and inducing systemic inflammation [17–19]. Although traditional views suggest that HPV primarily replicates locally with limited systemic effects, there are reports indicating that HPV DNA can be isolated from circulating white blood cells in both healthy blood donors and patients with HPV-associated cancers. This suggests that HPV may reach arterial structures via the bloodstream. [17] The role of chronic inflammation in promoting atherosclerotic plaque development is well-established, and HPV, particularly its high-risk strains, has been implicated in this process [7, 8]. Studies consistently demonstrate that women with high-risk HPV infections face an elevated risk of cardiovascular disease, even after adjusting for traditional risk factors such as smoking, physical activity, and BMI. The precise biological pathways remain unclear, but it is hypothesized that the chronic inflammation induced by HPV may accelerate the formation of atherosclerotic plaques and contribute to cardiovascular complications.

HPV’s role in impairing the function of the tumor suppressor protein p53 has been a key area of investigation. [20] The HPV E6 oncoprotein promotes the degradation of p53, disrupting its regulation of cell growth and repair, which may also contribute to the development of atherosclerotic plaques [21]. Reis et al. hypothesize that HPV infection may promote atheroma formation in patients by enhancing systemic inflammation or by directly targeting blood vessels through nucleic acids carried by extracellular vesicles, such as exosomes [22]. Additional research has highlighted a direct connection between the tumor suppressor protein p53 and the reprogramming of lipid metabolism, particularly in the regulation of non-cell-autonomous lysophospholipids. Mass spectrometry analysis revealed that the loss of p53 in pancreatic ductal adenocarcinoma (PDAC) cells leads to a significant reduction in lysophospholipids, sphinganine, and phosphatidylglycerol, which may influence the tumor microenvironment and immune response. [23] Although further research is needed, this mechanism could provide insight into how HPV-related p53 dysfunction contributes to cardiovascular disease. Additionally, studies found that HPV 16 can also affect vascular endothelium, where the E5 oncoprotein increases Vascular Endothelial Growth Factor (VEGF) expression by activating the EGFR, PI3K/Akt, and MEK/ERK1/2 pathways. Inhibitors targeting these pathways reduced VEGF expression, highlighting the role of E5 in promoting angiogenesis [24]. Additionally, there are studies suggesting that HPV infection may influence stroke pathogenesis through circRNA-mediated mechanisms. [25]

In the United States, the FDA approved the quadrivalent HPV vaccine (Gardasil) in 2006 and the bivalent HPV vaccine (Cervarix) in 2009. Gardasil is for females aged 9–26, while Cervarix is for those aged 10–25. Both vaccines target HPV types 16 and 18, which are strongly linked to cervical cancer, with Gardasil also covering types 6 and 11. Concerns about vaccine side effects are common, and studies have explored the link between HPV vaccination and rare cardiac conditions, with molecular mimicry as a potential cause. Kanduc identified 34 HPV pentamers resembling human proteins linked to arrhythmias, including nine related to Titin, a key muscle protein. Disruptions in Titin can lead to ventricular cardiomyopathy and sudden cardiac death. Other matches involve desmosomal proteins, associated with arrhythmogenic cardiomyopathy and increased risk of sudden death during sleep [26]. However, our study did not find a higher incidence of cardiovascular events in the vaccinated group. Nonetheless, more research is focusing on whether vaccines may play a protective role in cardiovascular health. A cross-sectional study analyzed data from 9353 women aged 20 to 59 from the 2003–2016 National Health and Nutrition Examination Survey (NHANES), all of whom underwent vaginal HPV DNA testing. After adjusting for sociodemographic factors, lifestyle, medical history, and family history of cardiovascular disease, vaginal HPV infection was associated with a higher risk of cardiovascular conditions such as coronary artery disease, heart attack, angina, and stroke (OR = 1.54, 95% CI 1.15–2.08). This association was not observed in vaccinated women (OR = 0.50, 95% CI 0.07–3.51) but remained significant in unvaccinated women (OR = 1.63, 95% CI 1.18–2.25) [14]. Although direct clinical evidence connecting HPV and these heart issues is limited, these findings highlight the need for more research into the cardiovascular effects of HPV and its vaccination.

This study presents several strengths. First, it utilized a large, nationwide cohort from the global TriNetX database, ensuring a diverse and representative sample. The use of PSM effectively minimized selection bias, allowing for better comparability between the HPV-vaccinated and non-vaccinated groups. Additionally, the long follow-up period of up to three years provided a comprehensive assessment of the long-term effects of HPV vaccination on cardiovascular and cerebrovascular outcomes. The inclusion of participants from various demographic and socioeconomic backgrounds further enhances the generalizability of the findings. Robust sensitivity analyses across different follow-up periods support the reliability of the results. Furthermore, it is one of the few studies that included male participants, broadening the scope of its conclusions. The detailed electronic medical record data also contributed to the precision of the study’s analysis.

This study has several limitations. While an association was observed, causality cannot be established due to the retrospective design and potential residual confounding. The study population was limited to individuals engaged with the U.S. healthcare system, which may introduce selection bias and limit generalizability. Although we included a 3-year follow-up, cardiovascular and cerebrovascular diseases may require longer periods to fully manifest; thus, extended longitudinal studies are warranted.

Data on HPV infection status, genotypes, and vaccination adherence (e.g., number of doses) were unavailable, limiting our ability to assess strain-specific protection or dose-dependent effects. The study focused on individuals aged 20–40 and did not include broader age groups or nonbinary gender identities, which may affect generalizability. Broad outcome categories such as “cardiovascular disease” may mask differences across distinct clinical conditions; future studies should explore individual outcomes such as myocardial infarction or stroke. Furthermore, although biological mechanisms were hypothesized, no direct biomarker or mechanistic validation was performed. The study period coincided with the COVID-19 pandemic, during which both infection and vaccination may have significantly influenced cardiovascular outcomes through inflammatory and hypercoagulable pathways. [27, 28] The impact of unmeasured factors, including family history, diet, exercise, COVID-19 infection and vaccination status, may also influence results. These limitations should be considered when interpreting our findings.

Future research should address key areas to clarify the link between HPV vaccination and cardiovascular health. Studies are needed to explore the biological mechanisms, particularly the roles of chronic inflammation and viral proteins like E6 and E7 in atherosclerosis. Comparative research on different HPV vaccine formulations, such as Gardasil and Cervarix, could optimize vaccine recommendations. Long-term surveillance of vaccinated populations is essential to evaluate sustained cardiovascular benefits. Investigating high-risk groups, including those with comorbidities, may reveal enhanced protective effects. Clinicians and public health policymakers may consider implementing targeted vaccination campaigns for individuals with elevated cardiovascular risk, such as those with metabolic syndrome or chronic inflammatory conditions. Extended follow-up of these populations may help determine the long-term cardiovascular effects and inform cost-effective immunization strategies. Finally, expanding research to diverse regions and demographic groups will help assess the findings’ generalizability and inform global public health strategies.

## Conclusion

In this large retrospective cohort study using a real-world US database, HPV vaccination was associated with a lower incidence of new-onset cardiovascular and cerebrovascular diseases. Subgroup analyses demonstrated consistent trends across sex, age, and racial groups; however, not all associations achieved statistical significance. These findings suggest a potential association between HPV vaccination and reduced cardiovascular risk, which warrants confirmation in prospective studies with detailed immunization and clinical data, as well as investigation into potential underlying biological mechanisms.

## Supplementary Information

Additional file1

## Data Availability

The data that support the findings of this study are available from the TriNetX Analytics Network. https://trinetx.com.

## Abbreviations

ASCVD: Atherosclerotic Cardiovascular Disease
BMI: Body Mass Index
CAD: Coronary Artery Disease
CI: Confidence Interval
CKD: Chronic Kidney Disease
CPT: Current Procedural Terminology
CVD: Cardiovascular Disease
EGFR: Epidermal Growth Factor Receptor
FDA: Food and Drug Administration
HIV: Human Immunodeficiency Virus
HPV: Human Papillomavirus
HR: Hazard Ratio
ICD-10: International Classification of Diseases, 10th Revision
IHD: Ischemic Heart Disease
IRB: Institutional Review Board
LOINC: Logical Observation Identifiers Names and Codes
MMPs: Matrix Metalloproteinases
NHANES: National Health and Nutrition Examination Survey
OR: Odds Ratio
PDAC: Pancreatic Ductal Adenocarcinoma
PI3K/Akt: Phosphatidylinositol-3-Kinase/Protein Kinase B Pathway
PSM: Propensity Score Matching
SMD: Standardized Mean Difference
TIMPs: Tissue Inhibitors of Metalloproteinases
VEGF: Vascular Endothelial Growth Factor
