# Supplementary material for: The association between HPV vaccination and new-onset cardiovascular and cerebrovascular diseases: based on a retrospective study
Source: J Health Popul Nutr. 2025 May 19;44:162. doi: 10.1186/s41043-025-00918-y (PMC12090540; doi:10.1186/s41043-025-00918-y)
Supplement: Supplementary file 1 — Additional file1 [file 41043_2025_918_MOESM1_ESM.docx]

**Table S1: Codes for definitions of covariates, outcomes**

| **Variable** | **Code(s)** |
| --- | --- |
| HPV Vaccine Codes | 90651: Nonavalent HPV vaccine  90650: Bivalent HPV vaccine  90649: Quadrivalent HPV vaccine |
| Cardiovascular Diseases | I20-I25: Ischemic heart diseases  I21: Acute myocardial infarction (MI)  I22: Subsequent MI (STEMI and NSTEMI)  I27: Other pulmonary heart diseases  I30-I52: Other forms of heart disease (deprecated 2021)  I50: Heart failure  I51.7: Cardiomegaly  2003: Left Ventricular Ejection Fraction (LVEF) ≤ 50% |
| Cerebrovascular Diseases | G45: Transient cerebral ischemic attacks and related syndromes  I67.2: Cerebral atherosclerosis  I63: Cerebral infarction  I61: Nontraumatic intracerebral hemorrhage  I62: Other and unspecified nontraumatic intracranial hemorrhage  I67: Other cerebrovascular diseases  I60: Nontraumatic subarachnoid hemorrhage  I60-I69: Cerebrovascular diseases (general category) |
| Heart Dysfunction | I50: Heart failure  I51.7: Cardiomegaly  2003: Left Ventricular Ejection Fraction (LVEF) ≤ 50% |

**Table S2: Sensitivity analysis for the risk of outcomes**

Follow for 5 years after the Index Date, (n=57,782)

| **Outcome** | **Cohort** | | |
| --- | --- | --- | --- |
|  | Patients with outcome | Survival Probability at End of Time Window | HR (95% CI) |
| 1. **Cardiovascular Diseases** |  |  |  |
| HPV vaccine | 2432 | 91.883% | 0.902 (0.854,0.952) |
| Non-HPV vaccine | 2815 | 91.303% | Reference |
| 1. **Cerebrovascular Diseases** |  |  |  |
| HPV vaccine | 164 | 99.302% | 0.71 (0.583,0.866) |
| Non-HPV vaccine | 244 | 99.256% | Reference |
| 1. **Heart Dysfunction** |  |  |  |
| HPV vaccine | 362 | 98.606% | 0.856 (0.745,0.983) |
| Non-HPV vaccine | 449 | 98.37% | Reference |
| 1. **Composite outcome** |  |  |  |
| HPV vaccine | 2542 | 91.431% | 0.886 (0.841,0.934) |
| Non-HPV vaccine | 2993 | 90.808% | Reference |

Hazard ratio (HR) and 95% CI are provided.
